# “In Silico” Characterization of 3-Phytase A and 3-Phytase B from* Aspergillus niger*

**DOI:** 10.1155/2017/9746191

**Published:** 2017-11-20

**Authors:** Doris C. Niño-Gómez, Claudia M. Rivera-Hoyos, Edwin D. Morales-Álvarez, Edgar A. Reyes-Montaño, Nury E. Vargas-Alejo, Ingrid N. Ramírez-Casallas, Kübra Erkan Türkmen, Homero Sáenz-Suárez, José A. Sáenz-Moreno, Raúl A. Poutou-Piñales, Janneth González-Santos, Azucena Arévalo-Galvis

**Affiliations:** ^1^Laboratorio de Biotecnología Molecular, Grupo de Biotecnología Ambiental e Industrial (GBAI), Departamento de Microbiología, Facultad de Ciencias, Pontificia Universidad Javeriana, Bogotá, Colombia; ^2^Departamento de Química, Facultad de Ciencias Exactas y Naturales, Universidad de Caldas, Manizales, Caldas, Colombia; ^3^Grupo de Investigación en Proteínas, Departamento de Química, Facultad de Ciencias, Universidad Nacional de Colombia (UNAL), Bogotá, Colombia; ^4^Department of Biology, Faculty of Science, Hacettepe University, Beytepe, Ankara, Turkey; ^5^Unidad de Biología Celular y Microscopía, Decanato de Ciencias de la Salud, Universidad Centroccidental Lisandro Alvarado, Barquisimeto, Venezuela; ^6^Grupo de Bioquímica Computacional y Estructural, Departamento de Bioquímica y Nutrición, Facultad de Ciencias, Pontificia Universidad Javeriana, Bogotá, Colombia; ^7^Laboratorio de Microbiología Especial, Grupo de Enfermedades Infecciosas, Departamento de Microbiología, Facultad de Ciencias, Pontificia Universidad Javeriana, Bogotá, Colombia

## Abstract

Phytases are used for feeding monogastric animals, because they hydrolyze phytic acid generating inorganic phosphate.* Aspergillus niger* 3-phytase A (PDB: 3K4Q) and 3-phytase B (PDB: 1QFX) were characterized using bioinformatic tools. Results showed that both enzymes have highly conserved catalytic pockets, supporting their classification as histidine acid phosphatases. 2D structures consist of 43% alpha-helix, 12% beta-sheet, and 45% others and 38% alpha-helix, 12% beta-sheet, and 50% others, respectively, and pI 4.94 and 4.60, aliphatic index 72.25 and 70.26 and average hydrophobicity of −0,304 and −0.330, respectively, suggesting aqueous media interaction. Glycosylation and glycation sites allowed detecting zones that can affect folding and biological activity, suggesting fragmentation. Docking showed that** H**_**59**_ and** H**_**63**_ act as nucleophiles and that** D**_**339**_ and** D**_**319**_ are proton donor residues. MW of 3K4Q (48.84 kDa) and 1QFX (50.78 kDa) is similar; 1QFX forms homodimers which will originate homotetramers with several catalytic center accessible to the ligand. 3K4Q is less stable (instability index 45.41) than 1QFX (instability index 33.66), but the estimated lifespan for 3K4Q is superior. Van der Waals interactions generate hydrogen bonds between the active center and O_2_ or H of the phytic acid phosphate groups, providing greater stability to these temporal molecular interactions.

## 1. Introduction

Most of the phosphorus (P) present in terrestrial ecosystems is located in the soil. Globally, the terrestrial biota contains 2.6 × 10^6^ g P, which is less than that contained in the soil, which oscillates between 96 and 160 × 10^6^ g P [1]. The highest transference of P from soil to biota occurs through the synthesis of organic compounds containing phosphorus (P) in plants, animals, and microorganisms. The organic compounds containing P are diverse, and their mineralization in the soil allows the P to be recycled back to the biota [1].

Phosphorus is an essential nutrient, which is involved in several biological functions such as regulation of intra- and extracellular pH, accumulation of energy in the form of ATP, lipid transport, and formation of biological membranes [2, 3]. Several compounds with organic phosphorus (oP) have different rates of mineralization. For example, oP from microorganisms (predominantly nucleic acids, 30–50% P in RNA and 5–10% P in DNA) and phospholipids (<10% P) is easily mineralized in soil environments [1]. However, other compounds with oP are not easily mineralized and can accumulate in the soil in substantial amounts. The most significant of these compounds is phytic acid (myo-inositol 1, 2, 3, 4, 5, 6 hexakisphosphate), [1].

Phytic acid is the main form of P storage in cereals, pulses, oilseeds, and nuts and constitutes 1–5% of its dry weight. In forage, one-third of the phosphorus is present as digestible inorganic phosphorus (iP), while the remaining two-thirds are present as oP in the form of phytates [4]. Phytates are a mixture of salts resulting from the union of phytic acid with divalent metal ions such as: Calcium (Ca^2+^), Copper (Cu^2+^), Iron (Fe^2+^), Magnesium (Mg^2+^), Manganese (Mn^2+^), and Zinc (Zn^2+^). Phytic acid can be bound to two different metals such as Calcium (Ca^2+^) and Magnesium (Mg^2+^), the resulting mixed salt is called phytin [4].

Phytate constitutes 65–80% of total P in grains and up to 80% of total P in manures of monogastric animals. Due to its negative charge, phytate is strongly adsorbed to various soil components once it is released from plant residues or manure [1].

On the other hand, the accumulation of phytate in the soil is due to the low possibility of being hydrolyzed by the phytase enzymes (E.C. 3.1.3.8), since the phytate dephosphorylation requires the binding of free phytate to the binding pocket of the substrate in the phytase enzyme. Thus, if phytate is tightly bound with soil components, it is not susceptible to be hydrolyzed by enzymes [1].

P from phytate is largely unavailable for monogastric animals, such as pigs and birds, due to the absence or the insufficient amount of phytase enzymes in the gastrointestinal tract to degrade it [5]; in this way it passes without being digested through the gastrointestinal tract. Since phytic acid can not be reabsorbed, feed for pigs and poultry is commonly supplemented with iP in order to meet the requirement of P, which increases production costs [6].

Supplementation with iP, along with the P from phytate excreted by monogastric animals, generates global ecological problems (eutrophication) as the discharge into rivers of wastewater with a high content of phytates results in the proliferation of cyanobacteria, hypoxia, and death of animals from aquatic environments [5]. The P present in phytate that is excreted in the manure of monogastric animals subsequently extends to farmlands, which often contributes to the eutrophication of surface waters, particularly in the areas of intensive livestock of pigs [7].

However, the adverse environmental and nutritional consequences of the presence of phytate in the diet of monogastric animals can be improved by the inclusion of phytases (E.C. 3.1.3.8) in their diet [5]. These enzymes are considered as an environmentally friendly product because (i) they reduce the amount of phosphorus entering the ecosystem, (ii) they reduce the problems caused by eutrophication of water, and (iii) they reduce the constant chelation or sequestration of nutritional factors in the soil, as well as in the digestive tract of poultry and pigs [8]. Phytases are produced by a wide variety of plants, bacteria, fungi, and yeasts. A commercial pair of phytases from the genus Aspergillus (Natuphos® and Ronozyme®) are currently available, as these filamentous fungi are the most prolific extracellular producers of this enzyme [7].

Some studies have shown that microbial sources are more promising for commercial phytase production. Although several strains of bacteria, yeasts, and fungi have been used for production under different conditions, two species,* A. niger* and* A. ficuum,* have been used more frequently for commercial phytase production [9]. Among the best known commercial phytases is found “Natu-phos” (Gist-Brocades NV Company, Netherlands). Natu-phos is a recombinant phytase produced by the expression of the* phy*A gene of* A. ficuum *NRRL 3135 in* A. niger* CBS 513.88, produced in 1994 [4, 7, 9].

In countries like Colombia and Venezuela, there is no legislation regulating the incorporation of phytase enzymes into the feed of monogastric animals, aimed at improving the bioavailability of phosphorus from the diet itself and at the reduction of the amount of phytate excreted in the feces. Therefore, the “in silico” analysis of physicochemical and structural properties, as well as the molecular docking analysis between the enzymes and the ligand, will allow researchers to gather information that is useful for the heterologous expression of the recombinant enzymes.

## 2. Materials and Methods

### 2.1. Protein Analysis

The phytases reported until September 13, 2015, were analyzed in the UNIPROT database (The UniProt Consortium) [10]. The PSI-Blast alignment [11] was performed between the amino acid sequences of the phytase reported for* A. niger*, which allowed determining its percentage of similarity and a multiple alignment with the ClustalO programs to identify conserved sites among the selected phytases. The ClustalW alignment allowed comparing the sequences of the two revised phytases: the 3-phytase A and the chains A and B of the 3-phytase B (http://www.ebi.ac.uk) [12].

### 2.2. Bioinformatic Analysis of the Reported and Revised Phytase from* A. Niger*

For this analysis, two protein structures resolved by X-ray crystallography (revised proteins) were used for* A. niger*: 3-phytase A and 3-phytase B. The primary sequences of the revised phytases were obtained from UniProtKB, Entry: P34755 and P34752, respectively, while tertiary structures were obtained from Protein Data Bank (PDB) [13], using the ID: 3K4Q and 1QFX, respectively.

### 2.3. Physicochemical Properties

The physicochemical properties of the amino acid sequences of the revised* A. niger* proteins were evaluated using the following programs: ProtParam and ProtScale [14] from ExPASy (http://www.expasy.org). The size of the window for the analyses with ProtScale was the basic nine amino acids recommended by the programs to ensure optimum coverage of the sequence when the path is made over it. In the case of the hydrophobicity profile, the Kyte and Doolittle algorithm was used, whose scale considers values between −4.5 and 4.5. The 3D structures of phytases from* A. niger* were visualized using the PyMOL (The PyMOL Molecular Graphics System, Version 1.8 Schrödinger, LLC) program.

### 2.4. Prediction of N-Glycosylation Sites

For the analysis of potential N-glycosylation sites in both phytases, the NetNGlyc 1.0 software [15] (http://www.cbs.dtu.dk) was used.

### 2.5. Prediction of Glycation Sites

For the analysis of the potential glycation sites in both phytases, Netglycate 1.0 software was used [16]. In addition, visualization of the 3D structures and determination of the distances between the *ε*-NH_2_ groups of the lysines and the side chains of Glutamate acid residues (E) and Aspartate (D) or basic residues of histidine (H), Arginine (R), and lysine (K) were performed using the SPDB-viewer 4.01 program [17, 18].

### 2.6. Prediction of Antigenic Peptides

Prediction of antigenic peptides in both proteins was performed by means of the antigenic peptide prediction tool (http://imed.med.ucm.es/Tools/antigenic.pl) [19] of the Complutense University of Madrid.

### 2.7. Ligand and Molecular Coupling Models (Rigid Docking)

The construction of the ligand (phytic acid) was performed using the Spartan version 4.0 molecular modeling program (https://www.wavefun.com/products/spartan.html), which has a graphical interface that allows the construction of the molecule atom by atom, selecting the appropriate hybridization according to the binding site of each element, followed by a minimization of the energy of the generated model.

The molecular coupling models of the reviewed phytases from* A. niger* against the phytic acid as a ligand were performed using the Autodock program [20] and the 3D structure of the ligand (phytic acid) was obtained with the Spartan program (before performing simulations of coupling with Autodock 4.2) [20]. The pocket containing the amino acids that form the highly conserved catalytic center in the revised phytase (histidine acid phosphatases) from* A. niger* (RHGXRXP-D) was identified from reports in the literature [21, 22]. Polar hydrogens were added to each receptor; the grid was located in the pocket of the active site for each model. Phytic acid boxes had the following dimensions and coordinates respectively: for 3-phytase A: X36, Y36 and Z38 (*x*: −6.396, *y*: 8.301 and *z*: 27.885), and for 3-phytase B: X36, Y34 and Z38 (*x*: 23.968, *y*: 71.06 and *z*: 69.576), with a space in both proteins of 0.375 Å. The network parameters and atomic affinity maps were calculated using AutoGrid 4 [20]. Each coupling simulation was carried out using the Lamarckian genetic algorithm with 2.500.000 energetic evaluations with a population of 150. Finally, the best poses of the ligand were determined based on the results of energy interaction given in kcal/mol.

## 3. Results

### 3.1. Computational Characterization

Until September 13, 2015, there were 12651 phytases in UNIPROT of which 115 (0.90%) belonged to* Aspergillus* spp., of which, 33 (28.69%) belonged to* A. niger*. For the genus Aspergillus, 11 phytases in total were reported as revised or cured; the distribution was as follows: one for each species* A. ficuum*,* A. oryzae*,* A. fumigatus,* and* A. nidulans*; two for* A. niger* and* A. awamori*, and three for* A. terreus*. The only two (6.06%) of the revised phytases of* A. niger* were 3-phytase A (E.C. 3.1.3.8, PDB ID: 3K4Q), corresponding to a monomer, and the 3-phytase B (E.C. 3.1.3.8, PDB ID: 1QFX), a homodimer.

**Figure 1:**
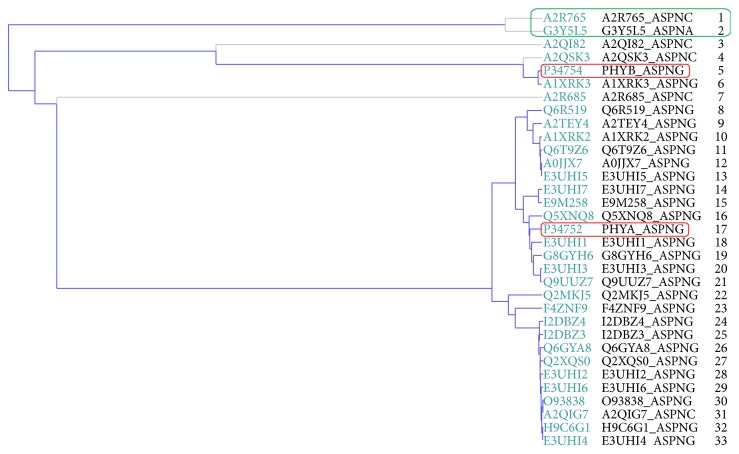
*Dendrogram of the 33 phytases reported for A. niger*. The first two phytases that appear are phytases that do not have PDB code or are not characterized within UNIPROT and are far from the two revised phytases. Alignment parameters are predetermined. The default transition matrix is Gonnet; the gap of the opening is 6 bits; the extension interval is of 1 bit. Clustal-Omega uses the HHafign algorithm and its default configuration as its core alignment engine [23].

The dendrogram between the 33 phytases reported for* A. niger* was obtained using the BLAST tool, and it was found that 3-phytase A (number 17, P34752) is closely related to 78.8% (26/33) of phytases whereas 3-phytase B (number 5, P34754) is only related to 9.1% (3/33) (Figure 1).

**Figure 2:**
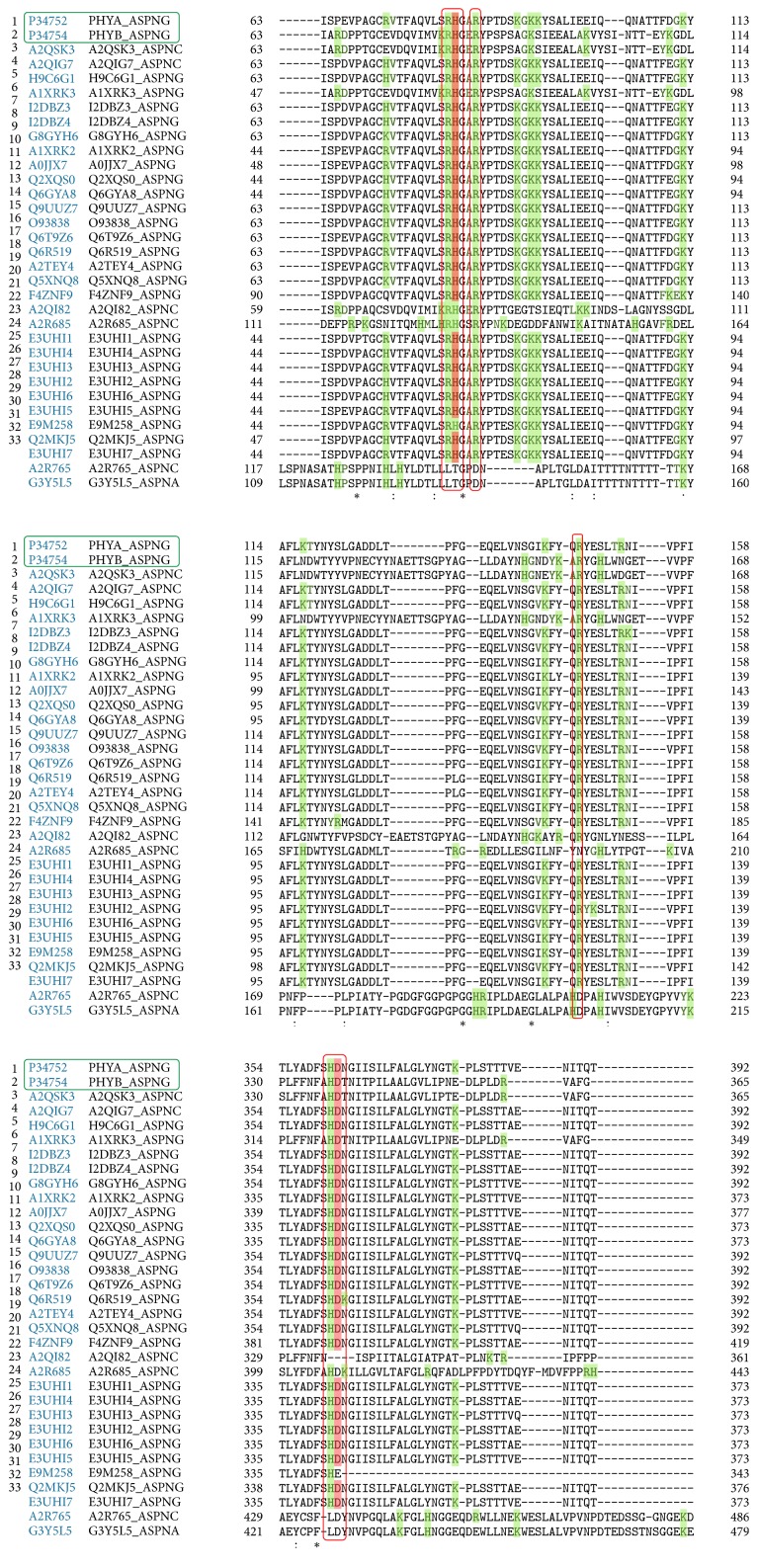
*Multiple alignment (Clustal O) among the 33 phytases reported for A. niger*. The green boxes indicate the two phytases reviewed and the red boxes indicate the amino acids that are highly conserved in the active site. The letters shaded in green correspond to positively charged amino acids.

The multiple alignment (Clustal O) between the amino acid sequences of the phytases reported for* A. niger* enabled the identification of a highly conserved sequence (R**H**GXRXP-H**D**) present in the histidine acid phosphatase family, corresponding to the pocket where the active ligand binding site is located (Figure 2).

### 3.2. Structural Characteristics of the Two Reported and Revised Phytases from* A. Niger*

The first revised phytase from* A. niger*, the 3-phytase A (PDB ID: 3K4Q), corresponds to a monomer. The second revised phytase, the 3-phytase B (PDB ID: 1QFX), initially corresponds to a homodimer (Chains A and B) which, thanks to its crystallographic symmetry, generates a homotetramer from two dimers.

When performing an alignment (Clustal W) between the sequences of the two chains of the initially dimeric protein (A and B) and the unique sequence of the monomeric protein, it was possible to determine that the chains A and B of the dimer are identical to each other, but different to the monomeric phytase.

### 3.3. 2D Structures of the Two Reported and Revised Phytases from* A. Niger*

**Figure 3:**
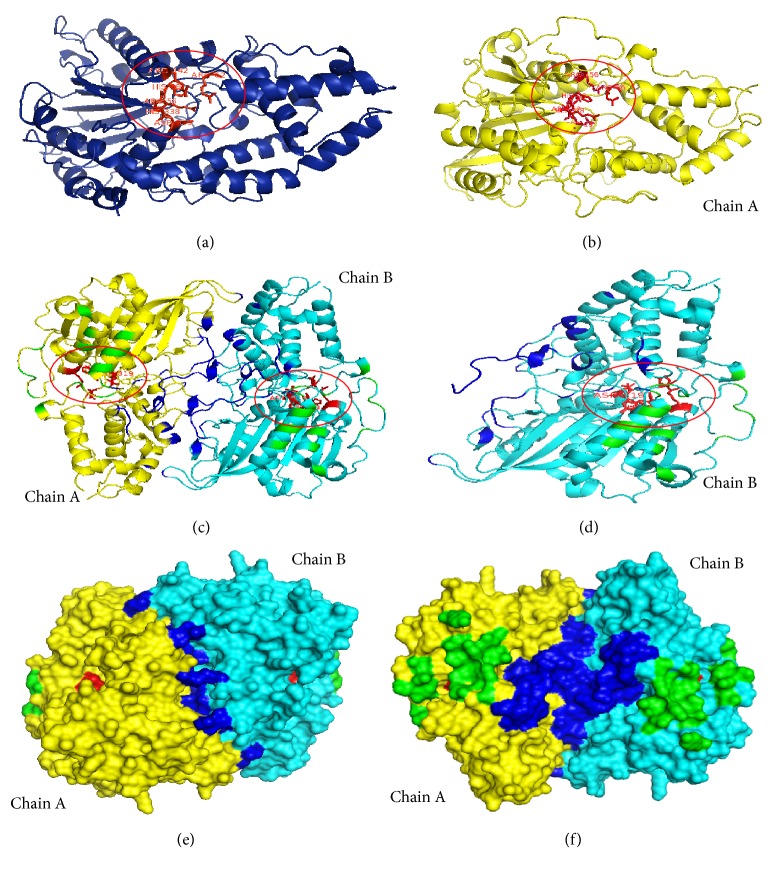
(a)* Ribbon diagram of 3-phytase A (PDB ID: 3K4Q)*. The 6 residues marked in red color form part of the pocket of the highly conserved ligand binding active site (R_58_, **H**_59_, G, X(R_62_), R_142_, X, P, H_338_, **D**_339_); (b)* ribbon diagram of the 3-phytase B chain A (PDBID: 1QFX)*; the 6 residues indicated in red color form part of the pocket of the highly conserved ligand binding active site (R_62_, **H**_63_, G, X(R_66_), R_156_, X, P, H_318_, and **D**_319_); (c) and (d)* active site rear view of the amino acids involved in the interactions that allow dimerization* (king blue color), tetramerization (green color), and active ligand binding sites (red color) in the dimer (left) and in the B chain of the 3-phytase B dimer (right); (e)* surface diagram* of the 3-phytase B top view; (f)* surface diagram* of the 3-phytase B bottom view.

The 3-phytase A (monomer) (Figure 3) (PDB ID: 3K4Q) has a 2D structure formed by 43% alpha helices, 12% beta sheets, and 45% random coils. A and B chains of 3-phytase B initially form a homodimer (Figure 3) (PDB ID: 1QFX) and its 2D structure corresponds to 38% alpha helices, 12% beta sheets, and 50% random coils. For the 3-phytase A, the active site is conformed by amino acids R_58_, **H**_59_, R_62_, R_142_, and **D**_339_ [22] (Figure 3(b)), and for the 3-phytase B the active site is conformed by amino acids R_62_, **H**_63_, R_66_, R_156_, H_318_, and **D**_319_ [21] (Figures 3(c) and 3(d)).

### 3.4. Structure of the Homodimer and the Tetramer Formed by Chains A and B of the 3-Phytase B from* A. Niger*

3-phytase B is initially a homodimer consisting of two identical chains A and B. The 39 amino acids that allow the interactions that lead to dimerization between the A and B chains are: Lys_14_-Tyr_24_, Leu_27_-His_29_, Tyr_36_, Glu_38_, Ser_41_-Ala_45_, Tyr_120_, Lys_217_, Leu_248_, Pro_252_-Ser_254_, Gln_262_-Asp_263_, Val_266_-Ser_267_, Asn_335_, Arg_342_, Phe_345_-Gly_346_, Ala_372_, Asp_393_, Gly_399_, Tyr_400_. The crystallographic symmetry generates a tetramer from the two dimers, and 17 amino acids that are involved in the interactions that allow the tetramerization of the protein have been identified: Cys_109_, Glu_114_, Thr_116_, Gly_118_, Ala_121_, Leu_123_-Leu_124_, Tyr_127_-Asn_128_, Asn_131_, Lys_163_, Glu_166_, Tyr_171_, Arg_447_, Pro_450_-Ile_451_, and Cys_453_ [21] (Figures 3(e) and 3(f)).

In both phytases, the fact that the pocket of the active site is composed mostly of positively charged amino acids (H-histidine and R-Arginine) is highlighted.

### 3.5. Physicochemical Characterization of the Two Reported and Revised Phytases from* A. Niger*

The physicochemical properties of the two phytases reported and reviewed for* A. niger* and obtained by the ProtParam bioinformatics program are detailed in Table 1.

### 3.6. Hydrophobicity and Accessibility Profiles of the Two Reported and Revised Phytases from* A. niger*

**Figure 4:**
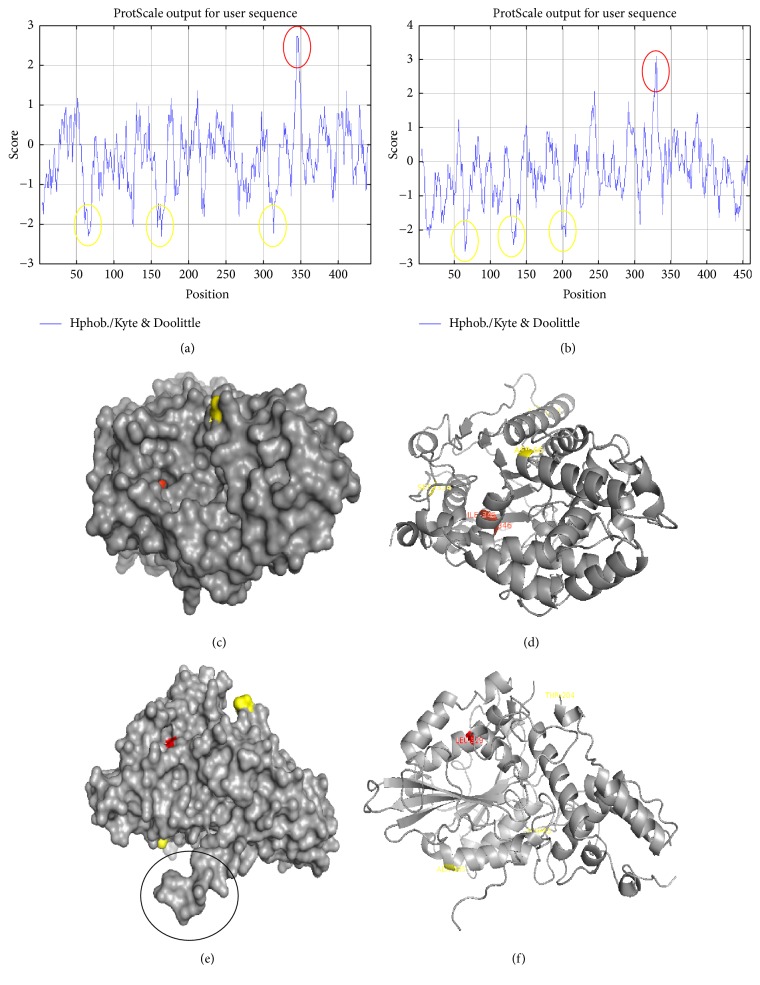
*Hydrophobicity profiles *(a and b) of phytases A and B from* A. niger*; (c) and (d) surface and ribbons diagram of the 3-phytase A; (e) and (f) surface and ribbons diagram of the chain A in the 3-phytase B, with the highest scoring amino acids (red color) and lower score (yellow color) of hydrophobicity. The circle in black color refers to the amino acids that allow homodimer formation in the 3-phytase B.

Figures 4(a) and 4(b) are the detailed hydrophobicity profiles of the phytases A and B from* A. niger*. The red circles represent the amino acids with a higher hydrophobicity score and the yellow circles represent the amino acids with a lower hydrophobicity score, according to the values recorded in Table 2.

**Figure 5:**
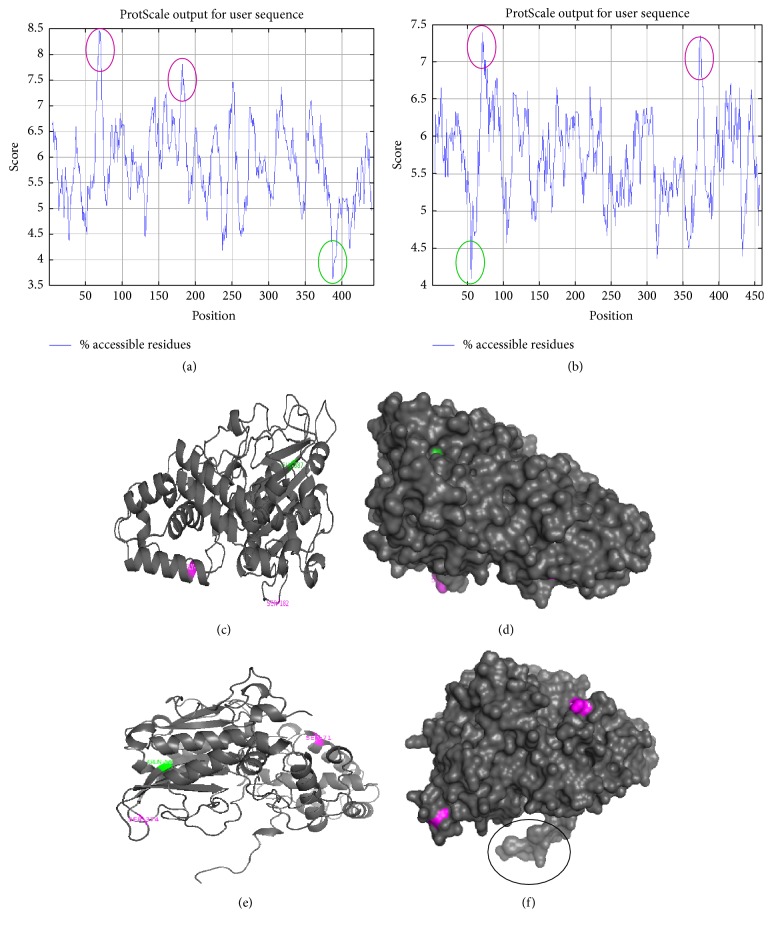
*Accessibility profiles* (a and b) of phytases A and B from* A. niger*. (c) and (d) Surface and ribbons diagram of the 3-phytase A. (e) and (f) Surface and ribbons diagram of chain A in 3-phytase B, where the amino acids with the minimum value (green color) and maximum value (purple) of accessibility are observed. The black colored circle refers to the amino acids that allow homodimer formation in 3-phytase B.

The accessibility profile of phytases A and B from* A. niger* is shown in Figure 5. Green colored circles represent amino acids with a minimum accessibility value and purple circles represent amino acids with a maximum accessibility value, according to the values recorded in Table 2.

### 3.7. Prediction of N-Glycosylation Sites in 3-Phytase A and Chain A of 3-Phytase B from* A. Niger*

Table 3 details the predictions of possible N-glycosylation sites of phytases A and B from* A. niger* by means of the NetNGlyc 1.0 tool, showing the position of the Asparagines (N), along both phytase chains that are located in an Asn-Xaa-Ser/Thr (where Xaa is any amino acid except proline) and for that reason could be glycosylated.

### 3.8. Prediction of Glycation Sites in 3-Phytase A and Chain A of 3-Phytase B from* A. Niger*

**Figure 6:**
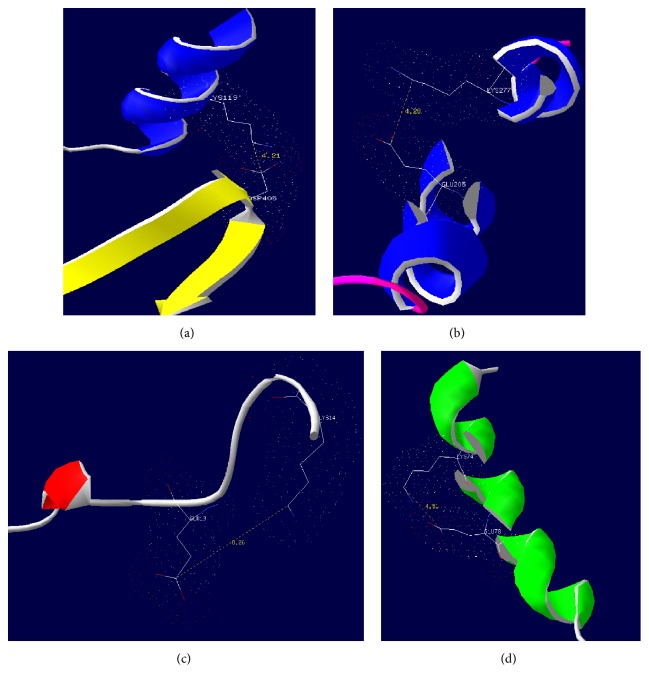
*Distances between amino acids involved in glycatio*n, Lysine_119_ (a) and Lysine_277_ (b), and acid residues (Asp_405_ and Glu_205_) in the 3D structure of 3-phytase A. Distances between Lysine_14_ (c) and the Lysine_74_ (d) and acid residues (Glu_19_ and Glu_78_) in the 3D structure of chain A of 3-phytase B.

In the case of 3-phytase A, Netglycate 1.0 predicted the glycation potential of seven lysines, whereas the methodology proposed by Sáenz et al., 2016 suggests the glycation of 14 of them. For Lys94, there is no prediction of glycation by either method.  Table 4 shows the comparison of the results and distances of the *ε*-NH2 groups of the lysines and the side chains of acidic or basic residues.  Figure 6 shows some distances between lysines and other acidic or basic residues in 3-phytase A.

In the case of 3-phytase A, Netglycate 1.0 allowed predicting the glycation potential of seven lysines along the protein sequence. When the glycation potential analysis was done by using the methodology proposed by Sáenz et al., 2016, the results suggest the glycation of 14 lysines. For Lys_217_, there is no prediction of glycation by either method.  Table 4 shows the comparison of the results and distances of the *ε*-NH_2_ groups of the lysines and acidic or basic residue side chains.  Figure 6 shows some distances between lysines and other acidic or basic residues in 3-phytase B.

### 3.9. Profile of Antigenicity of 3-Phytase A and Chain A of 3-Phytase B from* A. niger*

**Figure 7:**
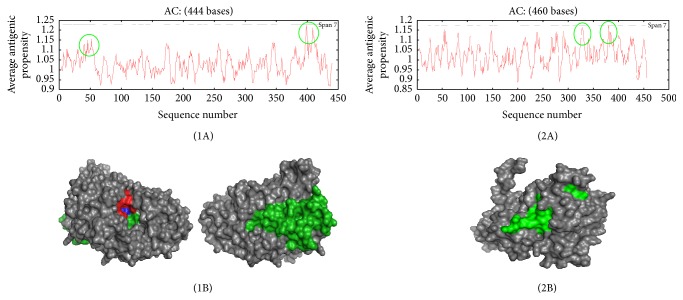
*Antigenicity profile*. (1A) 3-Phytase A from* A. niger*; (1B)* location of antigenicity peaks (green color)*. 3-Phytase A from* A. niger* and RHGXRXP-HD active site ($\underline{R_{58}}$, **H**_59_, R_62_, R_142_, H_338_, and **D**_339_); (2A) chain A in 3-phytase B from* A. niger*; (2B) l*ocation of antigenicity peaks (green color)*.

The antigenic propensity or average epitope of 3-phytase A is 1.0304; when the average for the complete protein is greater than 1.0 then all residues having more than 1.0 are potentially antigenic.  Figure 7(1A) shows the peaks of antigenicity in green colored circles along the chain of amino acids in 3-phytase A. In Figure 7(1B), the two peaks of antigenicity are located on the surface of the protein with higher score in green color and, additionally, its active center in red color is observed, corresponding to the information registered on the antigenic determinants in Table 5. The amino acid Arg_58_ forms part of the active site of the protein and at the same time is located in an area with a high antigenic propensity, highlighted in underlined font. The letters N highlighted in bold in Table 5 correspond to the positions of the Asparagines (N) which were identified as potential N-glycosylation sites and are also part of some identified antigenic determinant. This finding may be a contributing factor to the induction of an immune response by 3-phytase A.

The antigenic propensity or average epitope of chain A of 3-phytase B is 1.0234. In Figure 7(2A), the peaks of antigenicity in green circles are observed, along the chain of amino acids in chain A of 3-phytase B. In Figure 7(2B), the peaks of antigenicity in green color are located on the surface of the protein and additionally its active center is observed in red color, corresponding with the information registered on the antigenic determinants in Table 5; the amino acid Arg_156_ is a part of the active site of the protein and, at the same time, is located in an area with a low antigenic propensity, highlighted in underlined font. The letters N highlighted in bold in Table 6 correspond to the positions of the Asparagines (N) which were identified as potential N-glycosylation sites and are also part of some identified antigenic determinant. This finding may be a factor contributing to the induction of an immune response by 3-phytase B.

### 3.10. Ligand and Molecular Coupling Models (Rigid Docking)

**Figure 8:**
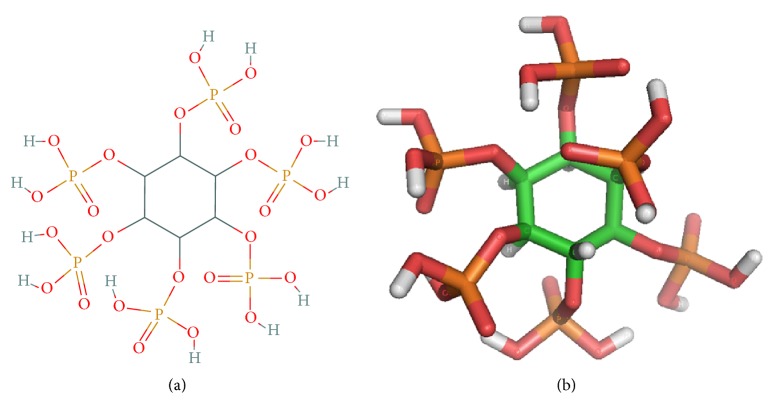
*3D structure of phytic acid (myo-inositol 1, 2, 3, 4, 5, 6 hexakisphosphate)*. (a) Diagram obtained from Pubchem; (b) 3D model generated by the Spartan 4.0 program and visualized with PyMOL.

The 3D structure of the phytic acid ligand (myo-inositol 1, 2, 3, 4, 5, 6 hexakisphosphate) was generated by the Spartan 4.0 program and is observed in Figure 8. This ligand was used in the molecular coupling models

**Figure 9:**
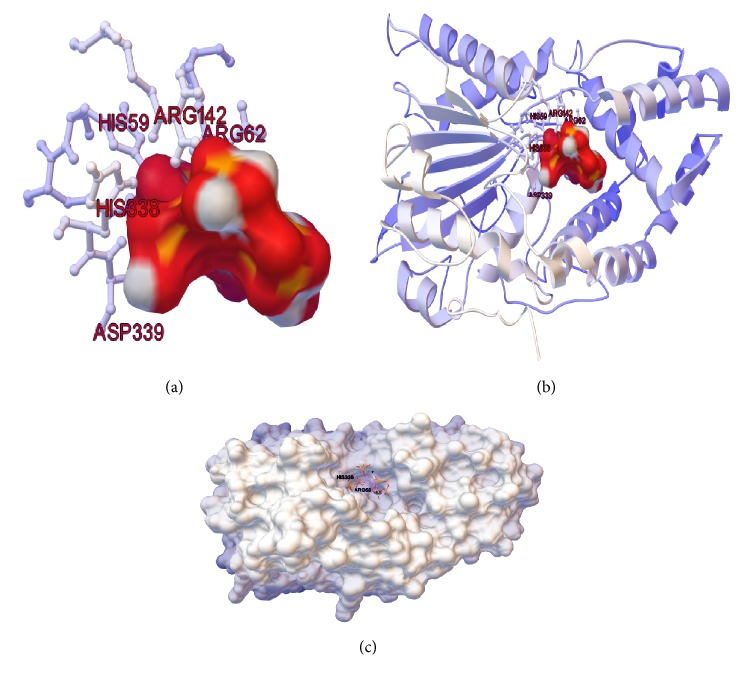
*Docking result between 3-phytase A (monomer) and phytic acid*. (a) Active site (RHGXRXP-HD) consisting of residues R_58_, **H**_59_, R_62_, R_142_, H_338_, and **D**_339_ versus phytic acid. (b) Ribbons diagram of the amino acids that make up the active site of the protein and (c) surface diagram of the phytic acid ligand attached to the pocket of the active site of the protein, visualized with the program Autodock.

**Figure 10:**
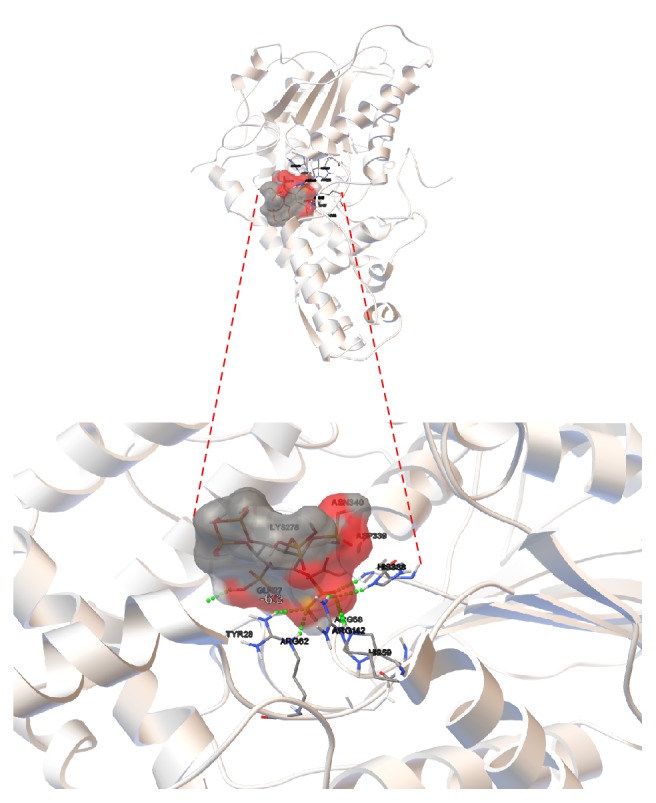
*General overview of the docking result between 3-phytase A and phytic acid (lowest energy = −6.3 kcal/mol)*. Active site (RHGXRXP-HD) consisting of residues R_58_, **H**_59_, R_62_, R_142_, H_338_, and **D**_339_ versus phytic acid, visualized with the Autodock program. The red colored areas in the phytic acid correspond to regions with negative charge. Green dots refer to the H Bridges established between the ligand and the amino acids that form the active site in the protein.

Molecular coupling models (Rigid Docking) were performed with the two revised phytases from* A. niger* and phytic acid as ligand which was directed to the catalytic pocket of the enzymes where the active site (R**H**GXRXP-H**D**) is located.  Figure 9 shows the result of the lower energy docking (−6.3 kcal/mol) between 3-phytase A and phytic acid.  Figure 10 provides an overview of the protein and the ligand coupling at the active site.

**Figure 11:**
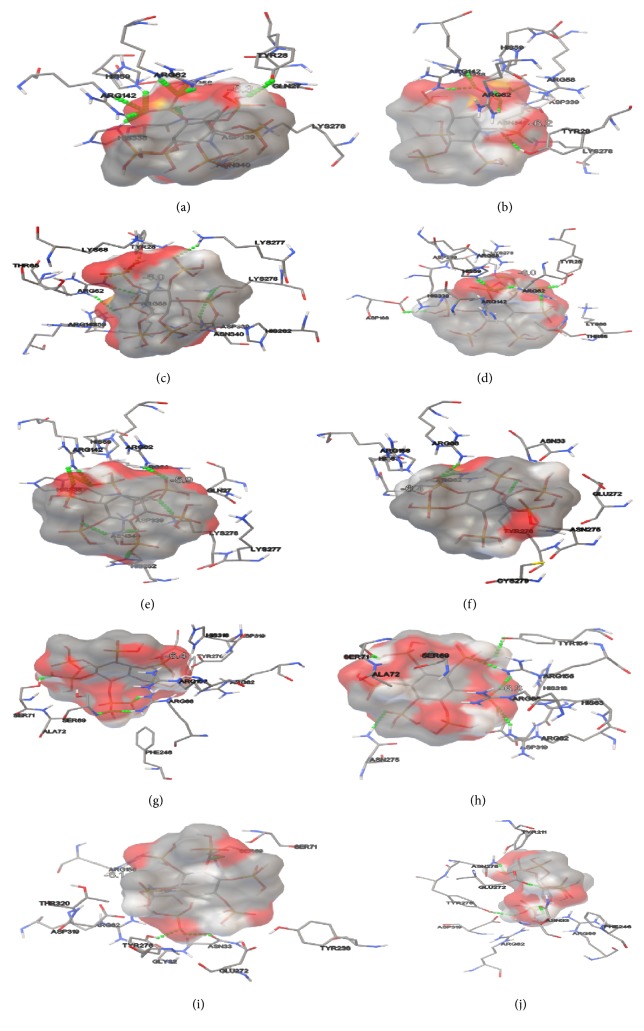
*Van der Waals electrostatic interactions involving the formation of hydrogen bonds (green dots)* between the active site amino acids and/or those closest to it in 3-phytase A (a–e), chain A of 3-phytase B (f–j), and the oxygens or hydrogens of the phytic acid ligand. Red colored regions can be seen, corresponding to negative regions in the electrostatic cloud of the ligand that make contact with the amino acids of the protein.

In Table 6, the results of the five lower docking energies (kcal/mol) obtained with the Autodock program at a distance of 0.375 Å are registered; the amino acids in bold refer to those that make up the highly conserved active site in the histidine acid phosphatases (HAP) and that are involved in the formation of hydrogen bonds with the ligand. In Figure 11, the results recorded in Table 6 are shown. The green dots in Figure 11 refer to the electrostatic interactions involving the formation of hydrogen bonds between the amino acids of the active site and/or the nearest ones to it, and the oxygens or hydrogens of the phytic acid ligand.

**Figure 12:**
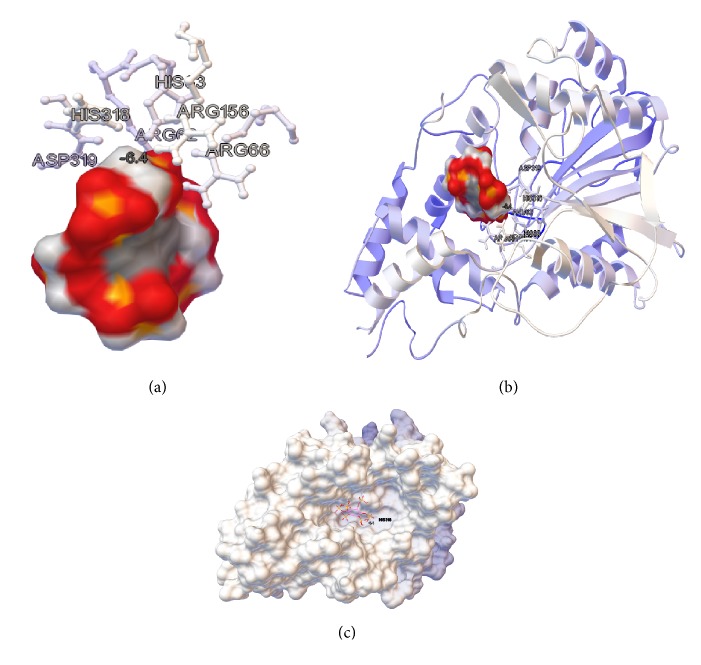
*Result of the docking between the A Chain of the homodimer from 3-phytase B and the phytic acid*. (a) Active site (R**H**GXRXP-H**D**) consisting of residues R_62_, **H**_63_, R_66_, R_156_, H_318_, and **D**_319_ versus phytic acid. (b) Ribbons diagram of the amino acids that compose the active site of the protein and (c) surface diagram of the phytic acid ligand attached to the pocket of the active site of the protein, visualized with the program Autodock.

**Figure 13:**
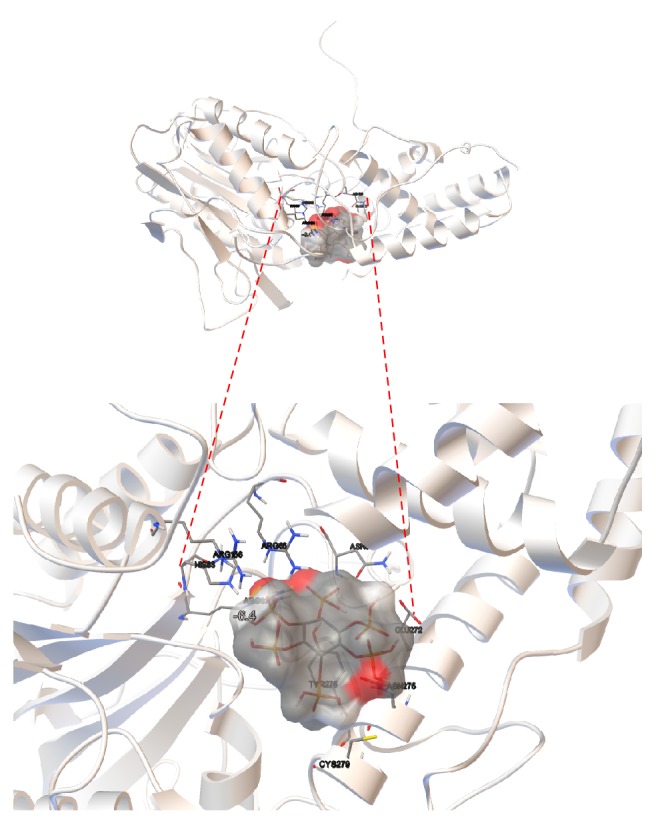
*General overview of the docking result between the A chain of the 3-phytase B homodimer and the phytic acid (lowest energy = −6.4 kcal/mol)*. Active site (R**H**GXRXP-H**D**) consisting of residues R_62_, **H**_63_, R_66_, R_156_, H_318_, and **D**_319_ versus phytic acid, visualized with the Autodock program. The red colored areas in the phytic acid correspond to regions with negative charge.

Figure 12 shows the docking result between the A chain of the 3-phytase B homodimer and the phytic acid. In Table 6, the results of the first five docking energies (kcal/mol) obtained with the Autodock program are shown.  Figure 13 provides an overview of the protein and the ligand coupling at the active site.

In Table 5, the results of the five lower docking energies (kcal/mol) obtained with the Autodock program at a distance of 0.375 Å are recorded; the amino acids in bold refer to those that make up the highly conserved active site in the histidine acid phosphatases (HAP) and that are involved in the formation of hydrogen bonds with the ligand. In Figure 12, the results recorded in Table 2 are evidenced. The green colored spots in Figure 11 refer to the electrostatic interactions involving the formation of hydrogen bonds between the amino acids of the active site and/or the closest ones to it and the oxygens or hydrogens of the phytic acid ligand.

## 4. Discussion

The database UNIPROT is characterized for being a nonredundant database, in which, on September 13, 2015, a large number of phytases was reported, 12651 in total, covering different species of microorganisms, plants, and animals. According to Casey and Walsh (2004), 115 (0.9%) phytases belonged to the Aspergillus genus, of which 33 (28.69%) corresponded to the species* A. niger*; they asserted that the genus Aspergillus is one of the most prolific extracellular producers of phytase enzymes [7].

Of these 33 reported phytases, 11 (33.3%) were found as revised for the genus Aspergillus, (phytases that were manually cured by experts in the UNIPROT database). Within this genus, the species* A. terreus* was the one that obtained a greater report of phytases, three in total. However, the three reports for this species corresponded to the same enzyme, 3-phytase A, expressed by the same* phy*A gene. In contrast, the* A. niger* species presented two reports of revised phytases, corresponding to different phytases, the 3-phytase A and the 3-phytase B, expressed by different* phy*A and* phy*B genes, respectively. Both revised phytases from* A. niger* were experimentally obtained by X-ray diffraction [21, 22].

Phytases A2R765 and G3Y5L5 in Figure 1 (green box) were removed from the computational analysis because they do not have PDB code or are not characterized within UNIPROT.

The ClustalW alignment showed that the sequences of these two phytases reviewed, the 3-phytase A with PDB ID: 3K4Q and the 3-phytase B with PDB ID: 1QFX, are different, being from different proteins. However, the sequences of the chains A and B that form the dimer of 3-phytase B are identical (homodimer). The first revised phytase of* A. niger*, the 3-phytase A, corresponds to a monomer and the second phytase revised, the 3-phytase B, corresponds to a homodimer formed by chains A and B. The two revised phytases from* A. niger* also differ in the amount and type of amino acids that form the signal peptide, being 23 residues for 3-phytase A and 19 for 3-phytase B.

The ClustalO alignment (Figure 2) showed that of the 33 reported phytases, 31 (93.9%) share a highly conserved motif corresponding to the active ligand binding site (RHGXRXP-HD) in the family of the histidine acid phosphatases “HAP” [22, 24, 25], which allows classification within this family. Within these 31 phytases, 3-phytases A and B (Figure 2, green box), revised phytases for* A. niger* species, were found. Phytases that were not characterized within the UNIPROT database (A2R765 and G3Y5L5), which were not assigned PDB code, did not present this highly conserved motif. Initially, it was possible to detect that the majority of the amino acids forming this highly conserved active site correspond to positively charged residues (Figure 2, green shading).

The enzymes 3-phytase A and 3-phytase B, belonging to the histidine acid phosphatases family (HAP), have the same enzymatic code E.C.3.1.3.8 according to the database BRENDA, which corresponds to the enzymes with phosphohydrolase activity. These enzymes catalyze the phosphomonoester bonds of phytic acid, releasing orthophosphate and producing final derivatives such as inositol and inositol monophosphate, which have a lower capacity to bind to metals [25]. This family of phytases share the active site (R**H**GXRXP-H**D**), whose catalytic pockets are for 3-phytase A (R_58_, **H**_59_, G, X_62_, R_142_, X, P, H_338_, **D**_339_) [22] and for the 3-phytase B (R_62_, **H**_63_, G_66_, X_156_, R_318_, X, P, H_318_, **D**_319_) [21]; both enzymes have activity at acidic pH (2.5–6.0) and at temperatures between 40 and 60°C, have low substrate specificity, and are capable of hydrolyzing phytate up to inositol monophosphate (IP1), [26].

The 2D structure of the monomer (Figure 3) of the 3-phytase A and the A chain of the 3-phytase B homodimer (Figure 3) have very similar conformations, being in higher proportion random coils (45% and 50%, resp.) and alpha helices (43% and 38% resp.) in both proteins and in a lower proportion (12% in both proteins) (Figure 3) which provides a more compact structure to both proteins. However, 3-phytase A presents a 3D structure formed by a single domain containing 20 alpha helices and only 8 beta sheets, while the A chain of the homodimer has a more complex structure made up of two domains with the active site located in the interface (Figure 3, red color). The largest domain consists of 11 alpha helices and 8 beta sheets, and the smallest consists of only 10 alpha helices.

The 3-phytase B has two identical A and B chains, which have 39 amino acids (Lys_14_-Tyr_24_, Leu_27_-His_29_, Tyr_36_, Glu_38_, Ser_41_-Ala_45_, Tyr_120_, Lys_217_, Leu_248_, Pro_252_-Ser_254_, Gln_262_-Asp_263_, Val_266_-Ser_267_, Asn_335_, Arg_342_, Phe_345_-Gly_346_, Ala_372_, Asp_393_, Gly_399_, Tyr_400_) which allow the interactions that give rise to its dimerization (Figure 3). Kostrewa et al. (1999) obtained a crystal of this protein, formed by a tetramer from two homodimers due to its crystallographic symmetry, and identified 17 amino acids involved in the interactions that facilitate the formation of the tetramer, thus achieving greater stability: Cys_109_, Glu_114_, Thr_116_, Gly_118_, Ala_121_, Leu_123_-Leu_124_, Tyr_127_-Asn_128_, Asn_131_, Lys_163_, Glu_166_, Tyr_171_, Arg_447_, Pro_450_-Ile_451_, Cys_453_ (Figure 3).

Two N-acetylglucosamine residues, NAG472 and NAG473, from a chain of carbohydrates bound to Ans_172_ are involved in the formation of the homotetramer in the crystal. These carbohydrate chains do not represent the complete natural glycosylation, but result from partial deglycosylation. The main tetramerization contacts are located on the opposite side of the entrance to the active site, so that the four active sites of the tetramer are exposed to the solvent and are easily accessible to the substrate [21].

In Figure 3, the amino acids forming the highly conserved ligand binding site (R**H**GXRXP-H**D**) in both phytases are detailed, which had initially been determined to be mostly positively charged amino acids (Clustal O alignment), because the ligand “phytic acid” is a very negative organic molecule which, thanks to the presence of the 6 phosphate groups (PO_4_^−3^), each of them located in a carbon atom of the inositol ring, binds to this active site by electrostatic interactions of Van der Waals type that generate temporal molecular couplings [27].

The amino acids indicated in red in both phytases correspond to the amino acids that perform the nucleophilic attack, according to the catalytic mechanism proposed by Oh et al. (2004), who assert that the histidine residue in the highly conserved active site R**H**GXRXP- serves as a nucleophile in the formation of a covalent phosphohistidine intermediate, while the aspartic acid residue from the C-terminal in the conserved sequence H**D** serves as a proton donor to the oxygen atom of the cleavable phosphomonoester bond, generating myo-inositol monophosphate as the final product.

The amino acid H_338_ that appears in the legend of Figure 3 between question marks is part of the conserved HD sequence in 3-phytase A that was not reported by Oakley (2010), but that participates in ligand binding; in this research, this amino acid is proposed as a part of the highly conserved active site as observed in molecular docking performed through the Autodock program at a distance of 0.375 Å.

The results obtained by the ProtParam program (Table 1) demonstrated that the 3-phytase A has a length of 444 aa and has a molecular weight of 48.84 kDa. Chain A of 3-phytase B, on the other hand, has a total length of 460 aa in mature state and a molecular weight of 58.78 kDa. These proteins have a p*I* of 4.94 and 4.6, respectively, which allows them to be classified as acid phytases.

3-phytase A (monomer) has a higher instability index (45.41) compared to chain A of 3-phytase B, whose instability results are inferior (33.66), which allows to catalog the latest as a stable protein (<40 = stable, values >40 = unstable), possibly due to the fact that the initial formation of the homodimer between the identical chains A and B gives it greater stability. Additionally, the formation of five intrachain disulfide bonds in both chains allows them to stabilize their three-dimensional structure.

The half-life of a protein is a prediction of the time it takes half the concentration of a protein in a cell to degrade after its synthesis. The 3-phytase A, being a monomeric protein, has a longer lifespan (>20 hours in yeast,* in vivo*) possibly because the N-terminal group in its sequence is Alanine (Ala1) and the proteins that possess Met, Ser, Ala, Thr, Val, or Gly at the N-terminal position register lifespans greater than 20 hours [28, 29].

On the contrary, chain A of 3-phytase B yielded a result of 3 minutes of lifespan in yeast, possibly because the N-terminal group in its sequence is phenyl alanine (Phe_1_) and the proteins that have Phe, Leu, Asp, Lys, or Arg at the N-terminal position register lifespans of less than 3 minutes [28, 29]. It appears that this factor involves the ubiquitin system, which is a small protein (76 amino acids) found in all eukaryotic cells and that undergoes an ATP-dependent reaction with proteins, condensing their C-terminal glycine residues with groups of amino of lysines of the protein to be labeled. These modified proteins are degraded shortly afterwards by a proteolytic complex which is recognized by the ubiquitin marker and because of this their lifespan is very short [28, 29]. The biological importance of the calculation of this parameter lies in the fact that the production of recombinant enzymes takes into account both the lifespan of the proteins to be expressed as well as their stability, in such way that a reduction of the degradation rate of proteins from heterologous genes is achieved.

The results of the aliphatic index of the two revised phytases from* A. niger,* 3-phytase A and 3-phytase B (72.25 and 70.46, resp.), allow the consideration of the fact that the relative volume occupied by its aliphatic side chains Ala, Val, Ile, and Leu increases thermostability in both phytases (both are globular secretory proteins).

The results of GRAVY (grand average of hydropathy) in 3-phytase A and 3-phytase B (−0.304 and −0.33, resp.) are obtained by combining the values of hydrophobicity and hydrophilicity of the side chains in their sequences. These negative values explain the reason why they tend to interact with aqueous media, typical of secretory proteins such as extracellular phytases [27].

The hydrophobicity profile allowed identifying that amino acids with a high score (Ile_345_ y Leu_346_ in 3-phytase A and Leu_329_ in 3-phytase B, Table 2) were found in areas with little exposure (inside alpha helices, Figures 4(c)–4(f)) in both proteins because such amino acids have aliphatic side chains that do not interact easily with aqueous solvents. On the contrary, amino acids with a minimum hydrophobicity score were found in exposed areas of the proteins (Ala_164_ in 3-phytase A and Glu_65_ in 3-phytase B). In the case of Glu_65_, being an amino acid whose R group does not have positive or negative charges at physiological pH, that is, pH close to 6.5 and 7.0, allows it to be solubilized more easily in aqueous solvents and, in the case of Ala_164_, to have a short aliphatic side chain that allows it to interact more easily with the aqueous medium. It should be noted that none of these amino acids were a part of the active site of ligand binding in both phytases. The amino acids indicated within the black circle in Figure 4(e) are involved in the formation of the homodimer in 3-phytase B and therefore are not found within an exposed zone of the protein. In general terms, 3-phytase A and 3-phytase B present few hydrophobic regions, as expected in secretory proteins [27].

The accessibility profile allowed the identification of the amino acids more or less exposed to the solvent, according to the score obtained and recorded in Table 2. For the 3-phytase A and the 3-phytase B, the Gly_69_ and the Ser_71_, respectively, were located in areas that were very exposed to the solvent and that were not a part of the active ligand binding site (Figures 5(c)–5(f)). The amino acid glycine has a simple structure, is the smallest amino acid, and is the only nonchiral amino acid, characteristics that allow it to acquire special conformations that other amino acids can not, and for this reason obtains a high solvent accessibility score. As for the serine amino acid, although it has an uncharged R polar group (-OH), it is short, very reactive, and hydrophilic, with a tendency to form hydrogen bonds with water.

The amino acids with the lowest accessibility score (Glu_387_ in 3-phytase A and Gln_56_ in 3-phytase B) were located inside the protein as a part of beta sheets (Figures 5(c)–5(f)) because glutamine is a polar amino acid and glutamic acid is negatively charged, which reduces its exposure to the solvent. According to the hydrophobicity profile, 3-phytase A and 3-phytase B present a high proportion of zones of easy accessibility to the aqueous medium along their sequences.

Secretory proteins, such as phytase enzymes, have carbohydrate addition or glycosylation sites that allow them to be recognized in the rough endoplasmic reticulum for their future correct folding and secretion [30, 31]. Using the NetNGlyc 1.0 bioinformatics program, 9 N-glycosylation sites for 3-phytase A (monomer) and 7 N-glycosylation sites for 3-phytase B (dimer) chain A were established. The positions of the Asparagines (N) along both phytase chains that were located in an Asn-Xaa-Ser/Thr section (where Xaa is any amino acid except proline) could be glycosylated (Table 3). It stands out that the seven possible N-glycosylation sites predicted for chain A of 3-phytase B must be duplicated because this phytase is formed by two identical chains, A and B, that initially form a homodimer; for that reason, it would have a total of 14 possible N-glycosylation sites for this phytase. The carbohydrate that binds directly to these N-glycosylation sites is normally* N*-acetylglucosamine [21, 22]. These added sugars will promote the correct folding of the phytases, deducing a mechanism of quality control of synthesis and assembly of the proteins, thus increasing its stability [32].

In glycation, the initial reversible reaction occurs between aldehyde or ketone groups of reducing sugars and *ε*-NH_2_ groups of lysines or the amino terminal of the protein. Subsequently, there is formation of Amadori products and finally AGEs* (Advanced Glycation End products)* are formed [33]. In general, the amino groups with the lowest p*K*a value should be more reactive towards glycation due to their nucleophilic capacity [34]. In the case of lysines, it has been suggested that the proximity of nearby residues plays a determining role to be or not glycated. The positively charged amino acids located near the primary structure or the three-dimensional structure decrease the p*K*a and thereby catalyze the glycation of such lysines [35]. Likewise, it has been suggested that the proximity of an acid residue to a lysine catalyzes the formation of Amadori products, which would make lysine more reactive to be glycated [36]. The results of the comparison of lysine prediction by the Netglycate algorithm [16], which exclusively considers the primary sequence of the protein and the methodology proposed by Sáenz et al. (2016), which uses the 3D structure of the protein, are shown in Table 4 and allow us to point out that, for the case of 3-phytase A, according to the proposal of the spatial relationship between the *ε*-NH_2_ group and side chains of acidic or basic residues as a requirement for glycation, 14 lysines with distances inferior to 9.89 are considered as potentially glycable. Although these distances exist in the 3D structure, only seven were considered by the algorithm Netglycate 1.0. The only lysine without glycation prediction by the two methodologies presented distances above 13.06 Å between the *ε*-NH_2_ group and the side chains of basic or acidic residues, a distance that would not allow the chemical interaction between these chemical groups as a requirement for glycation.

For the case of chain A of 3-phytase B, of the 10 lysines susceptible to being glycated as proposed by Sáenz et al. (2016), all with distances lower than 9.48 Å, only two are considered by Netglycate 1.0. The third lysine with prediction of glycation (K_413_) is not considered by this proposal since the group *ε*-NH_2_ is separated from the side chains of basic or acidic residues by distances greater than 23.84 Å, a distance that would not allow the chemical interaction between these chemical groups as a requirement for glycation. The only lysine not considered by the two methodologies is K_217_, whose *ε*-NH_2_ group is located away from the side chains of basic or acidic residues by distances greater than 13.15 or 11.97 Å, respectively, distances that would not allow the chemical interaction between these chemical groups as a requirement for glycation.

Ninety percent (9/10) of the Netglycate 1.0 predictions, as proposed by Sáenz et al. (2016), can be associated with the spatial relationship between lysines and acidic or basic residues less than 10 Å and the remaining 10% (1/10), the K_413_ of 1QFX, presents distances greater than 23.84 Å to acidic or basic residues even though, according to the data provided by the algorithm Netglycate 1.0, its probability (score) of occurrence of glycation is scarcely 59.8% (data not shown), being the lowest of all; however, it is predicted to be potentially glycable.

These results are in accordance with what Sáenz et al. (2016) proposed, because it is not necessarily the sequence (primary structure) but the spatial relationship in the 3D structure that favors the lysines glycation, provided that the distances of lysines to acidic or basic residues are less than 10 Å (Figure 6). In this sense, this type of enzymes with high percentages of acidic and basic residues and lysines close, in both the primary structure and the 3D structure, to acid residues or other basic residues generates a high probability of chemical interaction between that type of amino acids, required for glycation. Finally, the Netglycate algorithm only predicted as glycable lysines 37.5% of those proposed by Sáenz et al. (2016).

*“In vitro”* investigations with other proteins that have been brought into contact with reducing sugars show that glycation may affect biological activity [37]. Assays performed with recombinant human interferon-gamma (hIFN-*γ*) glycoprotein isolates in* E. coli* demonstrated that such purified protein was also prone to progressive proteolysis and covalent dimerization during storage, since late glycation stages cause the cleavage of the peptide bond and the covalent reticulation in lysine and arginine residues (but not of cysteine) [38]; that is to say that glycation promotes protein fragmentation and is produced in glycated lysines [17]. However, the* “in vitro”* effect caused in proteins should be studied carefully to correctly determine the cause-effect relationship, since the observed phenomena could be the consequence of the glycation of other components that can interact with the proteins or of the reactions between the protein and some by-product generated during glycation [37]. Therefore, the identification of these potential glycation sites in 3-phytase A and 3-phytase B chain A could represent potential sites of fragmentation of the concentrated or purified proteins during prolonged storage times, negatively affecting their biological activity.

In 3-phytase A, the average antigenic propensity was 1.0304, and when the average value is higher than 1.0, the amino acids that are above 1.0 will be potentially antigenic. According to the data recorded in Table 5 and compared to Figure 7(1A) (Green colored circles), two highly antigenic peaks or regions can be identified. The first region groups the amino acids from His_23_-Arg_58_, 36 amino acids in total including the amino acid Arg_58_, which is the first amino acid that forms part of the ligand binding active site and is therefore part of a solvent accessible zone. The second region registers the highest peak of antigenicity and integrates a greater amount of amino acids from Ser_374_-Arg_420_, 47 amino acids in total, being located in the opposite side to the active site and in a zone highly exposed to the solvent, as can be observed in Figure 7(1B). The prediction of antigenic peptides takes into account which peptide fragments of a protein are likely to be antigenic. These antigenic fragments should be located in solvent accessible regions and should contain hydrophobic and hydrophilic residues. Therefore, the second region which comprises the highest amount of amino acids and is completely exposed to the solvent would have a higher antigenicity.

In chain A of 3-phytase B, the average antigenic propensity is 1.0234. In contrast to 3-phytase A, the 3-phytase B chain A has more peaks or highly antigenic regions that cluster fewer amino acids; however, according to the data recorded in Table 5 and compared to Figure 7(2A) (green colored circles), two highly antigenic peaks or regions can be identified. The first region groups the amino acids Ile_322_-Glu_336_, 15 amino acids in total, located in an area highly exposed to the solvent. The second region contains amino acids Thr_338_-Asn_392_, 15 amino acids in total, but not all of them are exposed to the solvent. Five of these 15 are hydrophobic (Val_380_, Leu_382_, Val_383_, Leu_384_, and Val_388_) and therefore are located in the interior of the protein (Figure 7(2B)). Therefore, antigenic fragments of the first region, which are all located in solvent accessible regions and contain hydrophobic and hydrophilic residues, would exhibit greater antigenicity.

The positions of the Asparagines (N) that were identified as potential N-glycosylation sites and which are a part of the reported antigenic determinants are highlighted in bold in Table 5 because this is a factor that may contribute to the induction of an immune response by both phytases [39].

Considering the usefulness of phytases as a dietary supplement in monogastric animals, it is also pertinent to consider that the presence of regions with high antigenic propensity along the sequence of these proteins could be translated in the presence of allergens that could trigger an allergic reaction in the host, who ingests them. The process of digestion involves mechanical, chemical, and biochemical processes that allow macronutrients to be transformed into simpler molecules that can be absorbed and used by animals. But despite the fact that these digestive processes take place, significant amounts of protein from diets and that are immunologically active reach the intestinal mucosa of monogastric animals [40]. When there is an actual allergic reaction, the body produces antibodies (proteins that specifically bind to allergens to neutralize and remove them from the body). There are different types of antibodies, but the responsible for allergic reactions to food is known as immunoglobulin E (IgE). The IgE antibody binds to the allergens, triggering an allergic reaction. During this reaction, IgE activates the segregation of signaling molecules in the bloodstream, which simultaneously causes the common symptoms of food allergies such as skin rashes, inflammation, abdominal pain and inflammation, vomiting, and diarrhea [40]. However, in several investigations performed [41–43] in animals, no reports were found on allergic reactions provoked by phytases to the animals involved in the trials.

In the research conducted by Kostrewa et al. (1999) and Oakley (2010), the ligand used to obtain the crystalline structure of 3-phytase A and 3-phytase B, respectively, was myo-inositol-1,2,3,4,5,6-hexakis sulfate (IHS), a potent inhibitor of such enzymes. This chemical compound is isosteric and isoelectric with respect to myo-inositol 1, 2, 3, 4, 5, 6 hexakisphosphate (IHP) and is considered an excellent analogous substrate.

However, the ligand used in this investigation corresponded to the chemical compound myo-inositol 1, 2, 3, 4, 5, 6 hexakisphosphate (IHP), also called phytic acid, the main form of phosphorus storage in the cereals that make up the diet of monogastric animals. This chemical compound is highly negative due to the presence of 6 phosphate groups (PO_4_^−3^) in its inositol ring, which is why the active site of binding to this ligand in phytases is composed mainly of positively charged amino acids (R**H**GXRXP-H**D**), [21, 22].

The molecular coupling model (Rigid Docking) directed to the catalytic pocket of 3-phytase A formed by residues Arg_58_, **His**_59_, Arg_62_, Arg_142_, His_338_, and **Asp**_339_ [22], and phytic acid as ligand, yielded very interesting results that allowed establishing the formation of Van der Waals electrostatic interactions that generated hydrogen bonds between the amino acids that form the active site of the protein and the oxygen or hydrogen of the phosphate groups of phytic acid (Figure 11). The lowest 5 energies of the molecular coupling result were selected (Table 6) and it was possible to determine which amino acids were forming the hydrogen bonds.

Table 6 shows that in the case of 3-Phytase B there are 6 amino acids (Arg_58_, **His**_59_, Arg_62_, Arg_142_, His_338_, and **Asp**_339_) of the active center involved in the formation of 8 hydrogen bonds with phytic acid, generating the lower energy in docking (−6.3 kcal/mol), which is favorable because the greater number of hydrogen bonds formed between the active site of the enzyme and the ligand favors the stability of this temporary molecular interaction [27].

Research by Oakley (2010) reported that the amino acids that form the active site of the protein and therefore establish electrostatic Van der Waals type interactions with the analogous IHP ligand in the crystal by the X-ray diffraction method at a resolution of 2.20 Å are: Arg_58_, **His**_59_, Arg_62_, Arg_142_ and **Asp**_339_. However, the active site of the protein in histidine acid phosphatases presents highly conserved residues (R**H**GXRXP-H**D**), involving a histidine in the H**D** segment. By means of the molecular coupling (Rigid Docking) carried out in this investigation at a distance of 0.375 Å, it was possible to determine that the His_338_ is involved in the formation of a hydrogen bond with the phytic acid ligand that was not previously reported by Oakley (2010), but was consistent with the information reported in the PDB*sum* database [22].

For the molecular coupling model (Rigid Docking) directed to the catalytic pocket of chain A of 3-phytase B, formed by residues Arg_62_, **His**_63_, Arg_66_, Arg_156_, His_318_, and **Asp**_319_ [21] and phytic acid as ligand, the formation of Van der Waals electrostatic interactions that generated hydrogen bonds between the amino acids that form the active site of the protein and the oxygens or hydrogens from the phosphate groups of phytic acid was also detected (Figure 11). The lowest 5 energies of the molecular coupling result were selected (Table 6) and it was possible to determine which amino acids were forming the hydrogen bonds.

Table 6 shows that in the case of 3-Phytase B there are 6 amino acids (Arg_62_, Ser_71_, Try_154_, Arg_156_, Arg_156_, and Asn_275_) of the active center involved in the formation of 4 hydrogen bonds with phytic acid, generating the lower energy in docking (−6.3 kcal/mol).

It is interesting to note that Kostrewa et al., (1999) determined that the active site of 3-phytase B is subdivided into a catalytic center (R_62_, **H**_63_, R_66_, R_156_, H_318_ y **D**_319_) and a substrate specificity site (Asp_75_ and Glu_272_); however, only the amino acid Arg_66_ forms part of this active site and participates in the formation of the hydrogen bonds with the analogous substrate IHS in the crystal [21].

Table 6 shows the energy of docking number 2 (−6.4 Kcal/mol) which involves 3 of these amino acids (Arg_66_, Ser_69_ and Ser_71_); although they do not form a part of the active center of the protein, they do form hydrogen bonds with the analog substrate IHS in the crystal. In addition, they are reported in PDB*sum*. Therefore, taking into account the fact that the stability of this temporal molecular interaction depends mainly on the number of hydrogen bonds formed, the energy that would offer greater stability would be number 3, since it additionally involves a greater number of amino acids than those that are located in the active site of the enzyme [27].

## 5. Conclusions

The species* Aspergillus niger* expresses two phytases currently reported by the UNIPROT database: 3-phytase A (PDB ID: 3K4Q) corresponding to a monomer and 3-phytase B (PDB ID: 1QFX) corresponding to a homodimer (chains A and B) which, due to its crystallographic symmetry, generates a homotetramer from two dimers. These phytases have been crystallized and the genes encoding them (*phy*A and* phy*B gene, resp.) have been cloned and overexpressed in other microorganisms, which has allowed them to be widely used in the feed industry of monogastric animals. The computational characterization of the two phytases produced by* A. niger*, 3-phytase A and 3-phytase B, made it possible to establish that both phytases belong to the histidine acid phosphatases class, with the active ligand binding site (RHGXRXP-HD) highly conserved. The 3-phytase A and the 3-phytase B chain A possess a molecular length and a molecular weight that do not differ substantially, although the monomer is considered as an unstable protein and the homodimer has a shorter lifespan. The aliphatic index in both phytases allows to conclude that they are thermostable enzymes. The hydrophobicity profiles and accessibility showed that these phytases interact with aqueous media, which is a characteristic of secretory proteins. It was possible to identify possible glycosylation and glycation sites in both phytases, which could affect the correct folding of the proteins and their possible fragmentation during prolonged storage times and therefore their biological activity. Both 3-phytases, A and B, exhibited areas with high antigenic propensity which could affect the immune system of the animal that ingests them. Finally, the molecular coupling models in both phytases allowed verifying the formation of electrostatic interactions of Van der Waals type that generates hydrogen bonds between the amino acids that form the active center of the protein and the oxygens or hydrogens of the phosphate groups of phytic acid, providing greater stability to these temporary molecular interactions.

## Figures and Tables

**Table 1:** Physicochemical properties of the two reported and revised phytases from *A. niger*.

Physical and chemical properties	3-Phytase A (monomer)			3-Phytase B (chain A, Homodimer)		
(ProtParam)	PDB ID: 3K4Q			PDB ID: 1QFX		
aa sequence length	467			479		
Signal peptide length	23			19		
Mature protein length	444			460		

Molecular Weight (kDa)	48,84			58,78		

Instability Index	45,41 (Unstable)			33,66 (Stable)		

Disulfide bond	5 (Intrachain) in positions:			5 (Intrachain) in positions:		
	Cys_8_–Cys_17_			Cys_52_–Cys_368_		
	Cys_48_–Cys_391_			Cys_109_–Cys_453_		
	Cys_192_–Cys_442_			Cys_197_–Cys_422_		
	Cys_241_–Cys_259_			Cys_206_–Cys_279_		
	Cys_413_–Cys_421_			Cys_394_–Cys_402_		

Theoretical Isoelectric Point (iP)	4,94			4,6		

Estimated Lifetime	4,4 hours (Reticulocytes of mammals,* in vitro*)			1,1 hours (Reticulocytes of mammals,* in vitro*)		
	>20 hours (yeasts, *in vivo*)			3 minutes (yeasts,* in vivo*)		
	>10 hours (*E. coli, in vivo*)			2 minutes (*E. coli, in vivo*)		

Aliphatic Index	72,25			70,46		

Average Hydropathicity (GRAVY)	−0,304			−0,33		

Amino acids composition	Ala (A)	29	6.5%	Ala (A)	40	8.7%
	Arg (R)	19	4.3%	Arg (R)	14	3.0%
	Asn (N)	19	4.3%	Asn (N)	38	8.3%
	Asp (D)	29	6.5%	Asp (D)	24	5.2%
	Cys (C)	10	2.3%	Cys (C)	10	2.2%
	Gln (Q)	19	4.3%	Gln (Q)	14	3.0%
	Glu (E)	22	5.0%	Glu (E)	24	5.2%
	Gly (G)	30	6.8%	Gly (G)	37	8.0%
	His (H)	9	2.0%	His (H)	6	1.3%
	Ile (I)	18	4.1%	Ile (I)	19	4.1%
	Leu (L)	36	8.1%	Leu (L)	36	7.8%
	Lys (K)	15	3.4%	Lys (K)	13	2.8%
	Met (M)	4	0.9%	Met (M)	7	1.5%
	Phe (F)	25	5.6%	Phe (F)	20	4.3%
	Pro (P)	22	5.0%	Pro (P)	27	5.9%
	Ser (S)	49	11.0%	Ser (S)	34	7.4%
	Thr (T)	39	8.8%	Thr (T)	32	7.0%
	Trp (W)	4	0.9%	Trp (W)	6	1.3%
	Tyr (Y)	18	4.1%	Tyr (Y)	35	7.6%
	Val (V)	28	6.3%	Val (V)	24	5.2%
	Pyl (O)	0	0%	Pyl (O)	0	0%
	Sec (U)	0	0%	Sec (U)	0	0%

Total number (%) of negatively charged amino acids (Asp + Glu)	51 (11.48%)			48 (10.43%)		

Total number (%) of positively charged amino acids (Asp + Glu)	34 (7.65%)			27 (5.86%)		

**Table 2:** Hydrophobicity score in the two reported and revised phytases from *A. niger* (ProtScale). Minimum and maximum values of accessibility in the two reported and revised phytases of *A. niger* (ProtScale).

Physicochemical property	3-Phytase A (444 residues)		3-Phytase B (460 residues)	
	Residue position	Score	Residue position	Score
Hydrophobicity	Ala_164_	***−2.300 (min.)***	Glu_65_	***−2.633 (min.)***
	Asp_66_	***−2.289***	Ala_135_	***−2.200***
	Ser_314_	***−2.211***	Thr_204_	***−2.211***
	Ile_345_	**2.722 (max.)**	Leu_329_	**3.089 (max.)**
	Leu_346_	**2.722 (max.)**		

	Residue position	Value	Residue position	Value

Accessibility	Glu_387_	*3.633 (min.)*	Gln_56_	*4.089 (min.)*
	Ser_182_	**7.811**	Ser_374_	**7.344**
	Gly_69_	**8.467 (max.)**	Ser_71_	**7.389 (max.)**

**Table 3:** *Possible N-glycosylation sites*. Three phytases A and B from *A. niger*.

aa position		Potential	Jury agreement	N-Glyc
*Prediction of N-glycosylation sites for 3-phytase A (PDB ID: 3K4Q)*				
(1)	27 **N**QSS	0.5302	(6/9)	+
(2)	59 **N**ESV	0.6564	(9/9)	++
(3)	105 **N**ATT	0.6414	(7/9)	+
(4)	120 **N**YSL	0.7272	(9/9)	++
(5)	207 **N**NTL	0.5930	(7/9)	+
(6)	230 **N**FTA	0.6720	(8/9)	+
(7)	339 **N**HTL	0.4021	(7/9)	−
(8)	352 **N**STL	0.7211	(9/9)	++
(9)	376 **N**GTK	0.7904	(9/9)	+++
(10)	388 **N**ITQ	0.6418	(8/9)	+

*Prediction of N-glycosylation sites for Chain A of 3-phytase B (PDB ID: 1QFX)*				
(1)	87 **N**TTE	0.4822	(4/9)	−
(2)	172 **N**YST	0.6708	(8/9)	+
(3)	208 **N**LTY	0.7573	(9/9)	+++
(4)	231 **N**LTA	0.6870	(9/9)	++
(5)	296 **N**ASL	0.5727	(6/9)	+
(6)	321 **N**ITP	0.1572	(9/9)	−−−
(7)	406 **N**YTS	0.6257	(8/9)	+
(8)	423 **N**VSA	0.5649	(5/9)	+
(9)	439 **N**TTT	0.5323	(7/9)	+

**Table 4:** Distances between lysines and acidic or basic residues in the 3D structure of 3-phytase A and the chain A of 3-phytase B and their relation with the prediction of possible glycation sites.

Lysine position	Acid residue distance: Å	Basic residue distance: Å	Glycation prediction	
			Netglycate 1.0 [16]	[17]
*3-Phytase A (ID: 3K4Q)*				
68	Lys_68_-Glu_205_: 8.26	Lys_68_-Lys_70_: 6.51	X	X
70	Lys_70_-Asp_66_: 4.09	Lys_70_-Lys_71_: 9.05; Lys_70_-Lys_68_: 6.51		X
71	Lys_71_-Glu_233_: 9.89		X	X
89	Lys_89_-Asp_223_: 5.68		X	X
94	Acid residue location > 13.98	Basic residues location > 13.06	—	—
119	Lys_119_-Asp_405_: 4.21; Lys_119_-Asp_12_: 4.27			X
148		Lys_148_-Lys_149_: 9.73		X
149	Lys_149_-Glu_152_: 4.52		X	X
158	Lys_158_-Asp_161_: 7.65		X	X
160	Lys_160_-Asp_161_: 9.97		X	X
172	Lys_172_-Asp_174_: 4.50		X	X
254	Lys_254_-Asp_244_: 9.84			X
277	Lys_277_-Asp_239_: 7.30; Lys_277_-Asp_202_: 9.16; Lys_277_-Glu_205_: 4.28	Lys_277_-Lys_68_: 7.84; Lys_277_-Lys_278_: 6.72		X
278		Lys_278_-Lys_277_: 6.72; Lys_278_-His_282_: 6.51		X
356	Lys_356_-Asp_370_: 8.5; Lys_356_-Glu_364_: 9.83			X

*TOTAL*			*7*	*14*

* Chain A of 3-phytase B (ID: 1QFX)*				
14	Lys_14_-Glu_19_: 8.26		X	X
28	Lys_28_-Glu_38_: 8.55; Lys_28_-Asp_22_: 6.95	Lys_28_-His_29_: 8.6		X
61	Lys_61_-Asp_125_: 6.56	Lys_61_-His_360_: 4.63; Lys_61_-His_129_: 4.40		X
74	Lys_74_-Glu_77_: 9.04; Lys_74_-Glu_78_: 4.51; Lys_74_-Asp_75_: 5.95			X
82	Lys_82_-Glu_78_: 9.46; Lys_82_-Asp_236_: 4.15			X
92	Lys_92_-Glu_90_: 4.61			X
134		Lys_134_-His_139_: 9.21		X
163	Lys_163_-Glu_159_: 8.49; Lys_163_-Glu_166_: 4.74	Lys_163_-Arg_447_: 9.84		X
217	Acid residues location > 11.97	Basic residues location > 13.15	—	—
285	Lys_285_-Glu_284_: 7.35			X
307	Lys_307_-Glu_308_: 9.81		X	X
413	Acid residues location > 29.31	Basic residues location > 23.84	X	

*Total*			*3*	*10*

**Table 5:** Antigenic determinants of 3-phytase A and of chain A in 3-phytase B from *A. niger*.

Fragment number	Position	Sequence	Position	Total Number
	Initial		Final	a.a.
*Antigenic determinants of 3-phytase A (Long. Total = 444 a.a.)*				
*Mean antigenic propensity = 1.0304*				
1	23	*HLWGQYAPFFSLA * **N** *ESVISPEVPAGCRVTFAQVLSR*	58	36
2	374	*SAWTVPFASRLYVEMMQCQAEQEPLVRVLVNDRVVPLHGCPVDALGR*	420	47

*Antigenic determinants of chain A in 3-phytase B (Long. Total = 460 a.a.)*				
*Mean antigenic propensity = 1.0234*				
1	322	*ITPILAALGVLIPNE*	336	15
2	378	*TYVRLVLNEAVLPFN*	392	15

**Table 6:** Results of lower docking energies between 3-phytase A and the A chain of 3-phytase B versus phytic acid, obtained with the Autodock program.

Classification	Energy (kcal/mol)	Number of hydrogen bonds formed	AA involved
*Docking energy between 3-phytase A and phytic acid*			
First	−6.3	8	**A** **r** **g**_58_, **His**_59_, **Arg**_62_, **Arg**_62_, **Arg**_142_, **Arg**_142_, **His**_338_, **Asp**_339_
Second	−6.2	7	Tyr_28_, **Arg**_62_, **Arg**_62_, **Arg**_142_, **Arg**_142_, **His**_338_, **Asp**_339_
Third	−6.0	7	Tyr_28_, **Arg**_62_, **Arg**_62_, **Arg**_62_, Lys_277_, Lys_278_, Asn_340_
Fourth	−6.0	5	Tyr_28_, **His**_59_, **Arg**_62_, **Arg**_62_, Lys_278_
Fifth	−5.9	8	**A** **r** **g**_62_, **Arg**_142_, **Arg**_142_, Lys_278_, His_282_, **His**_338_, Asn_340_, Asn_340_

*Docking energy between 3-phytase B chain A and phytic acid*			
First	−6.4	3	**A** **r** **g**_62_, **Arg**_66_, Tyr_276_
Second	−6.4	4	**A** **r** **g**_66_, **Arg**_66_, Ser_69_, Ser_71_
Third	−6.3	6	**A** **r** **g**_62_, Ser_71_, Try_154_, **Arg**_156_, **Arg**_156_, Asn_275_
Fourth	−6.1	3	Asn_33_, Ser_69_, Tyr_276_
Fifth	−5.9	4	Asn_33_, Glu_272_, Asn_275_, Tyr_276_
